# Fascinating single‐cell red algae: models for evolution and adaptation

**DOI:** 10.1111/nph.71024

**Published:** 2026-02-18

**Authors:** Frédéric Berger, Debashish Bhattacharya, Chung Hyun Cho, Seok‐Wan Choi, Julia Van Etten, Shunsuke Hirooka, Tzu‐Yen Huang, Kyle J. Lauersen, Yongsung Lee, Shao‐Lun Liu, Shin‐ya Miyagishima, Stephen D. Rader, Daniel Schubert, Hwan Su Yoon

**Affiliations:** ^1^ Gregor Mendel Institute, Austrian Academy of Sciences, Vienna BioCenter Vienna 1030 Austria; ^2^ Department of Biochemistry and Microbiology Rutgers, The State University of New Jersey New Brunswick NJ 08901 USA; ^3^ Department of Biological Sciences Sungkyunkwan University Suwon 16419 Korea; ^4^ Biology Department Woods Hole Oceanographic Institution Woods Hole MA 02543 USA; ^5^ Biology Department University of Maryland College Park MD 20742 USA; ^6^ Department of Gene Function and Phenomics National Institute of Genetics 1111 Yata, Mishima Shizuoka 411‐8540 Japan; ^7^ International Graduate Degree Program for Biodiversity Tunghai University Taichung 40704 Taiwan; ^8^ Biodiversity Program Taiwan International Graduate Program Academia Sinica Taipei 11529 Taiwan; ^9^ Biological and Environmental Sciences and Engineering Division King Abdullah University of Science and Technology (KAUST) Thuwal 23955‐6900 Saudi Arabia; ^10^ Department of Chemistry and Biochemistry University of Northern British Columbia Prince George BC V2N 4Z9 Canada; ^11^ Epigenetics of Plants Freie Universität Berlin 14195 Berlin Germany

**Keywords:** biotechnology, Cyanidiophyceae, ecology, genomics, red algae

## Abstract

The unicellular red algae, Cyanidiophyceae, that diverged early during Archaeplastida (algal and plant) evolution, occupy a variety of extreme habitats that are inhospitable for most other eukaryotes. With the use of modern genomics and genetics methods, Cyanidiophyceae show a remarkable taxonomic diversity, share haplodiplophasic life cycles, and are engaged in complex trophic interactions with microbes that occupy geothermal niches. Amenable to molecular engineering, Cyanidiophyceae are excellent models for understanding evolutionary mechanisms that underpin their extremophilic lifestyles. Their unique growth conditions make these choice red algae of high interest for biotechnological exploitation in environments unsuitable for crops.

**Table:** 

	Contents	
	Summary	1424
I.	Introduction	1425
II.	Cyanidiophyceae systematics and evolution	1425
III.	Life cycle of Cyanidiophyceae	1426
IV.	Cyanidiophyceae ecology	1428
V.	Importance of Cyanidiophyceae in biotechnology	1430
VI.	Molecular tools	1432
VII.	Molecular responses to stress in Cyanidiophyceae	1432
VIII.	Conclusions	1433
	Acknowledgements	1433
	References	1434

## I. Introduction

After the first description of *Galdieria sulphuraria* in 1899 by the Italian scientist A. Galdieri (Galdieri, 1899), various members of the eukaryotic algal class Cyanidiophyceae have since been discovered and their classification has been continuously revised (Cho *et al*., 2023; Park *et al*., 2023; Huang *et al*., 2024). Cyanidiophyceae are dominant in extreme environments such as volcanic areas characterized by high temperatures (> 40°C), acidic waters (pH 0.5–4), and high concentrations of heavy metals (Rothschild & Mancinelli, 2001; Matsuzaki *et al*., 2004; Misumi *et al*., 2008). These algae have small genomes (< 20 Mbp, < 8000 genes) and two widely recognized extremophilic species, so far *Cyanidioschyzon merolae* and *G. sulphuraria*, can be engineered efficiently (Miyagishima & Tanaka, 2021; Hirooka *et al*., 2022). Their tolerance to a vast range of temperature, pH, and heavy‐metal concentrations minimizes microbial contaminants in their growth medium (Miyagishima & Tanaka, 2021) and provides opportunities to study mechanisms of detoxification and heat resistance. Beyond their fundamental biological significance, these models are also becoming a focus of research in biotechnology. Here, we review recent progress in the various aspects of research using these fascinating organisms.

## II. Cyanidiophyceae systematics and evolution

**Fig. 1:**
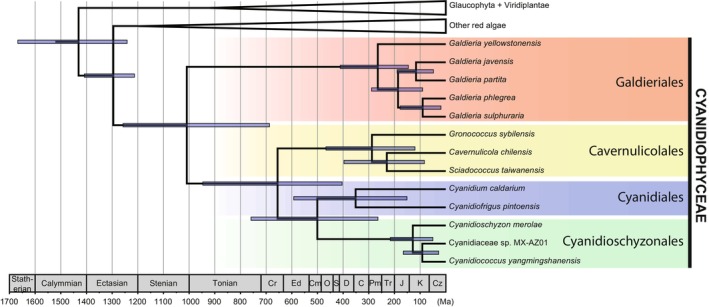
Bayesian time‐calibrated phylogeny of Cyanidiophyceae. Divergence time estimates were obtained using priors from a previous analysis (Yang *et al*., 2016), which was supplemented with additional genetic data from Cyanidiophyceae species. Bayesian divergence time estimation was conducted with BEAST v.2.6.6 (Bouckaert *et al*., 2019) using a mixed dataset comprising nucleotides (2623 sites) from two ribosomal genes (LSU, SSU) and amino acids (2054 sites) from five protein‐coding genes (*ef2*, *cox1*, *psa*A, *psb*A, *rbc*L). Geological Period and Era abbreviations: C, Carboniferous; Cm, Cambrian; Cr, Cryogenian; Cz, Cenozoic; D, Devonian, Ed, Ediacaran; J, Jurassic; K, Cretaceous; O, Ordovician; S, Silurian; Pm, Permian, Tr, Triassic.

Following the origin of all red algae *c*. 1.43 billion years ago (bya), the Cyanidiophyceae lineage diverged from other red algal groups *c*. 1.3 bya (Fig. 1), making it the earliest diverged branch within this phylum (Yoon *et al*., 2006). A recent high‐rank taxonomic revision has reclassified Cyanidiophyceae into four orders and four families based on plastid genome data: Galdieriales, Cavernulicolales, Cyanidiales, and Cyanidioschyzonales, whereby Cyanidiales is now restricted to a narrow lineage including *Cyanidium* rather than the entire class (Park *et al*., 2023). The diversification of the four orders within Cyanidiophyceae began *c*. 1 bya, which is contemporaneous with, or even precedes the divergence of other major crown‐group eukaryotes during the Tonian period (Xiao & Tang, 2018).

Galdieriales were the first order to diverge *c*. 1 bya and consist of a single genus, *Galdieria*, which includes six species that share similar genomic organization. Whereas their plastid genome is relatively similar to other Cyanidiophyceae, their mitochondrial genome shows reduced size and highly diverged sequences (Cho *et al*., 2020) that make it difficult to reconstruct a mitogenome phylogeny. Nuclear genomic features also distinguish this order from other Cyanidiophyceae. The small genome of these species (13–17 Mb) encompasses 50–60 chromosomes with frequent gene duplication in subtelomeric regions (Schönknecht *et al*., 2013; Rossoni *et al*., 2019a; Hirooka *et al*., 2022; Cho *et al*., 2023). The peculiar and fluctuating genome organization may enhance fitness in the extreme environments they inhabit via adaptive gene family expansion in the subtelomeric regions (Cho *et al*., 2023). Another notable characteristic is the versatile heterotrophic ability of Galdieriales, which can utilize > 36 carbon sources (Gross & Schnarrenberger, 1995; Masson *et al*., 2025). Diploid Galdieriales cells grow up to 10 μm and can form up to 32 autospores (De Luca *et al*., 1978; Merola *et al*., 1981; Albertano *et al*., 2000).

The other three orders (Cavernulicolales, Cyanidiales, and Cyanidioschyzonales) are similar in genome structure. Compared with Galdieriales, all published chromosome‐level genome assemblies from Cyanidiales and Cyanidioschyzonales converge into 20 contigs without exception, contain more intron‐less genes (Matsuzaki *et al*., 2004; Cho *et al*., 2023) and encode fewer protein coding genes (*c*. 5000 vs 7000–8000 in Galdieriales). No genome assemblies are available for Cavernulicolales (as of December 2025). These species also share traits likely to have been inherited from the common ancestor of these lineages such as the arsenic detoxification pathway (Cho *et al*., 2023), haploid cells that are usually 3–6 μm in diameter, and meiosis that results in 2–4 spores.

Cavernulicolales is a recently proposed order that is composed of so called ‘cave Cyanidium’. It is the earliest diverged order in this lineage, estimated at having split *c*. 650 million years ago (Ma) (Fig. 1). Subsequently, the divergence of Cyanidiales and Cyanidioschyzonales occurred *c*. 500 Ma. In contrast to Cyanidiales and Cyanidioschyzonales (Ciniglia *et al*., 2019), they possess a thick cell wall. Species in this order are not thermoacidophiles; rather, they inhabit humid coastal caves with dim light, mild temperature, and weak acidity (Azúa‐Bustos *et al*., 2009; Ciniglia *et al*., 2019). Due to this specificity, cell growth fails in either established media for thermophilic Cyanidiophyceae or conventional culture media for marine and freshwater algae. Consequently, no culture strains are available up to now. The neoproterozoic oxygenation event and increased oxygenation of volcanic emissions may have facilitated the transition of the lineage to mesophilic habitats (Laakso & Schrag, 2017). Further studies are required to elucidate possible mechanisms underlying this physiological adaptation (Box 1).

Box 1 **Outlook**

**What are the main challenges?**

Further studies of Cavernulicolales are required, and uni‐algal cultures made available to scientists to elucidate possible mechanisms underlying this physiological adaptation.

**Where do the authors think progress can be made/into which vein should efforts be channeled?**

Other geothermal locations in Iceland, Japan, the Middle East, and New Zealand may be home to undiscovered Cyanidiophyceae.

Increased efforts in biotechnological applications are warranted; *Galdieria* and *Cyanidioschyzon merolae* could be the algae that appropriately interface with industrial sites. Further genetic engineering of *Galdieria* for biotechnological application, including engineering its plastid genome, is to be shown. *Cyanidioschyzon merolae* would rather be a chassis for synthetic genomics applications.

**What are the main outstanding questions?**

The role of cell ploidy in adaptation to natural habitats, specifically how this trait reflects local selective pressures, needs to be further elucidated.

What is the underlying genetic architecture of species that inhabit neighboring extreme habitats (potentially cryptic species) and what does this teach us about local adaptation and the drivers of this process?

Future investigations of community interactions that involve Cyanidiophyceae to elucidate how they interact with other microbes and to better understand how the community as a whole is functioning/stabilizing as well as to gain insights into mechanisms of past HGT/adaptation.

By what mechanism has the GC content of *Galdieria* drifted from that of the other Cyanidiophyceae?

Place more interest in the mitochondria and plastidial genomes and more particularly to investigate coordination between the nuclear, plastid, and mitochondrial genomes.

**What are the potential generalizable biological insights?**

*Cyanidioschyzon merolae* is a valuable model system for streamlined molecular biology (e.g. splicing and stress responses) and should therefore continue to yield valuable discoveries about the ancestral state of these processes as well as on the minimal composition of components required to maintain functional processes.

Cyanidiales, once used widely to encompass various species in Cyanidiophyceae, now only include the two species, *Cyanidium caldarium* and *Cyanidiofrigus pintoensis*. The former has long been used in studies, whereas the latter species was recently described and prefers relatively lower temperatures (20–30°C) and nonaquatic microhabitats (Huang *et al*., 2024). The recent discovery of *Cyanidiofrigus pintoensis* points to the value of searching for nonthermophilic species of Cyanidiales that have not been identified using traditional approaches.

Cyanidioschyzonales includes *Cyanidiococcus yangmingshanensis* and *C. merolae*; the latter has become the model species to study Cyanidiophyceae because it can be genetically manipulated (Kuroiwa *et al*., 1994; Matsuzaki *et al*., 2004). Overlapping exons result in the smallest genome size of Cyanidiophyceae, and this might have accompanied adaptation to high temperatures (Park *et al*., 2023), which Cyanidioschyzonales best tolerate among Cyanidiophyceae (Huang *et al*., 2024).

## III. Life cycle of Cyanidiophyceae

**Fig. 2:**
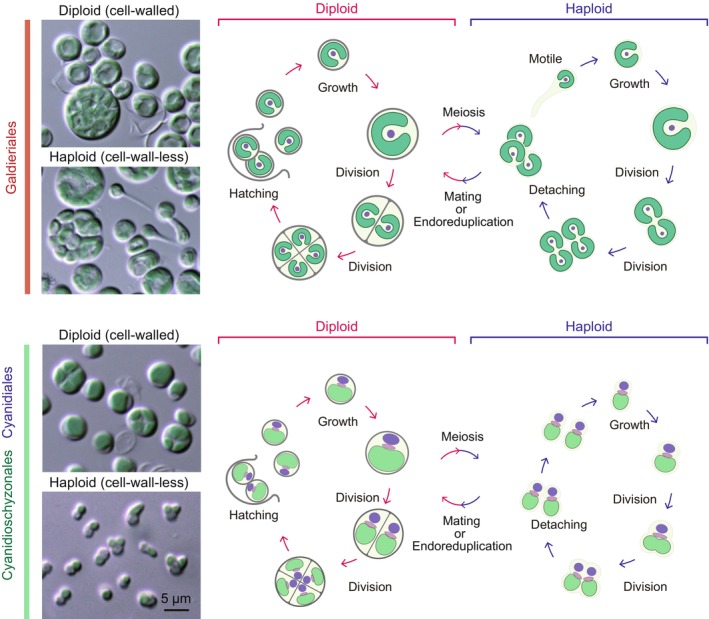
Life cycle of Cyanidiophyceae. The ‘default’ state found in natural habitats is the cell‐walled diploid phase. Specific signals (yet unknown) trigger meiosis, leading to transition to the haploid phase with two mating types. Acquisition of competence for mating triggers fertilization and resumes the diploid phase.

Since their first description (Galdieri, 1899), Cyanidiophyceae were believed to solely reproduce asexually although their genomes contain genes potentially involved in meiosis (Umen & Coelho, 2019). However, sexual reproduction has recently been discovered in these algae. In *Galdieria*, the natural and previously known cell‐walled form is diploid (Fig. 2) (Hirooka *et al*., 2022). When the pH of laboratory cultures is lowered from 2 to 1, these cells undergo meiosis and produce cell‐wall‐less haploids. As with diploids, haploids are also able to proliferate asexually by cell division. The haploids possess two mating types and undergo isogamy, in which morphologically indistinguishable gametes fuse to generate a diploid. In addition, a haploid can generate a diploid by endoreduplication in laboratory culture.

Regarding other members (genera) of the Cyanidiophyceae – namely *Cyanidioschyzon*, *Cyanidiococcus*, and *Cyanidium* – several lines of circumstantial evidence, as described below, suggest that previously unrecognized sexual reproduction also occurs in these genera, as in *Galdieria*. *Cyanidioschyzon merolae* found in Naples, Italy was first described as the sole cell‐wall‐less species in Cyanidiophyceae (De Luca *et al*., 1978), and *C. merolae* 10D has been used as a model organism in which procedures for genetic manipulation have been developed (Miyagishima & Tanaka, 2021). However, genetically closely related strains have been found in Yellowstone National Park, United States but these are cell‐walled and proliferate by forming four daughter cells through two successive cell divisions within the mother cell wall before release (Toplin *et al*., 2008; Lee *et al*., 2015), which is similar to *Cyanidiococcus* and *Cyanidium* (Jong *et al*., 2021). *Cyanidiococcus yangmingshanensis* was first described as a cell‐walled species that proliferates by forming four daughter cells within the mother cell wall, as in *Cyanidium* (Liu *et al*., 2020). However, in the same species but different strain, cells morphologically resembling *C. merolae* 10D and apparently lacking a cell wall have also been observed (Cho *et al*., 2020). These observations raise the possibility that the cell‐walled form producing four daughter cells within a mother cell represents the diploid phase, whereas the cell‐wall‐less form proliferating by binary fission corresponds to the haploid phase in these three genera.

Under these circumstances, our recent study demonstrated that cell‐walled forms of *Cyanidiococcus*, *Cyanidioschyzon*, and *Cyanidium* give rise to cell‐wall‐less forms when cultures are transferred to lower pH media. Cytological and genomic analyses indicate that the cell‐walled and cell‐wall‐less forms are diploid and haploid, respectively, and that both diploids and haploids can proliferate asexually, representing haplodiplontic life cycles (Fig. 2). Notably, the *ACTIN* gene (in the case of *Galdieria partida*, one of four *ACTIN* genes, *ACT3*) is specifically expressed in the diploid phase, where actin localizes at the cell division plane and is involved in cytokinesis, whereas cytokinesis in the cell‐wall‐less haploid phase occurs without actin (Hirooka *et al*., 2022).

Collectively, four genera of Cyanidiophyceae – *Galdieria*, *Cyanidioschyzon*, *Cyanidiococcus*, and *Cyanidium* – are naturally cell‐walled diploids that form four daughter cells within the mother cell wall through actin‐dependent cytokinesis. Under certain specific environmental stimuli (yet to be identified; although under laboratory conditions haploid cells were generated at low frequency when cultures were transferred to lower pH media), they give rise to isogamous, cell‐wall‐less haploids, which can also proliferate via actin‐independent cell division. Thus, *C. merolae* 10D is a haploid clone that was probably derived from an originally cell‐walled diploid (Fig. 2).

## IV. Cyanidiophyceae ecology

In the geothermal area, Cyanidioschyzonales and Galdieriales exhibit distinct preferences and relative abundances across geothermal microhabitats (Ciniglia *et al*., 2004; Skorupa *et al*., 2013; Hsieh *et al*., 2015). These habitats differ markedly in light intensity, temperature range, and organic carbon availability (Supporting Information Fig. S1; Table 1), enabling spatial differentiation and local adaptation of Cyanidiophyceae along broader environmental gradients. Environmental amplicon‐base surveys demonstrate that Cyanidioschyzonales dominate sunlit aquatic sites such as hot springs and streams, whereas Galdieriales prevail in darker, drier niches including sulfur vents, soils, and endolithic cavities (Ciniglia *et al*., 2004; Skorupa *et al*., 2013; Hsieh *et al*., 2015). This spatial separation reflects their physiology. Cyanidioschyzonales are obligate photoautotrophs tolerant of high temperature (Kobayashi *et al*., 2014; Huang *et al*., 2024) and irradiance (Fu & Wang, 2023) but are drought‐sensitive (Pinto *et al*., 2007); see also our drought simulation assay in Fig. S2. By contrast, Galdieriales are more desiccation‐tolerant (Fig. S2), acclimate to cold (Rossoni & Weber, 2019), and – thanks to prokaryote‐derived horizontal gene transfer (HGT) – use urea and grow mixotrophically/heterotrophically under terrestrial, low light conditions (Qiu *et al*., 2013; Rossoni *et al*., 2019a; Schönknecht *et al*., 2013; see Fig. S1 for likely allochthonous organic sources). Notably, the mixotrophic capacity of Galdieriales helps them withstand photodamage when subjected to higher light conditions (Fu *et al*., 2019). Together, these findings highlight how spatial differentiation between these two orders is closely linked to their distinct but flexible physiological traits. When organic carbon is available, earlier work showed that no mixotrophy, only heterotrophy, is observed in *G. sulphuraria* (strain 074G) (Oesterhelt *et al*., 2007). But more recent work showed that another strain of *G. sulphuraria* (strain SAG21.92) can grow mixotrophically (Curien *et al*., 2021). The former strain was later recognized to be *G. javensis*, a different species from the latter strain now differentiated as *G. partita* (Park *et al*., 2023; Huang *et al*., 2024). In a similar vein, recent work suggests that mixotrophic capacity varies among Galdieriales species and is influenced by the type of organic carbon available (Huang *et al*., 2024). Some species (*G. partita* when treated with glucose or glycerol, and *G. daedala* when treated with glycerol) exhibit enhanced growth under mixotrophic conditions compared to heterotrophy alone, whereas others (*G. >daedala* and *G. javensis* when treated with glucose) show little or no benefit. Notably, mixotrophic growth outperformed heterotrophy, highlighting the importance of carbon‐source specificity. Surprisingly, it has been found that elevated carbon dioxide acts as a trigger to promote heterotrophy in multiple *Galdieria* species. Stable isotope analysis was used to demonstrate this CO_2_ is not consumed by the cells in this state, but its absence can cause cessation of glucose consumption from the medium (Masson *et al*., 2025). These differences highlight the need for further investigation into the evolution of mixotrophy in this group and how it contributes to their adaptation to diverse and extreme environments (Box 1).

**Table 1:** Environmental characteristics of aquatic and nonaquatic habitats where Cyanidiophyceae biofilms were observed.

Features	Aquatic	Nonaquatic
Microhabitat	Hot springs, pools, streams	Soil, sulfur vents, endolithic, epilithic
Temperature	40–63.4°C	19–48°C
pH	1.5–2.6	0.4–1.2
DOC content	TOC in stream: 0.71 (μg C ml^−1^)	WEOC in soil: 32.25 (μg C g^−1^ dry soil)

DOC, dissolved organic carbon; TOC, total organic carbon; WEOC, water‐extractable organic carbon.

For aquatic samples (streams), DOC content is represented as TOC. For nonaquatic (soil) samples, it is expressed as WEOC. Data summarized from Hsieh *et al*. (2015).

Despite broad‐scale spatial differentiation, temporal niche complementarity allows these orders to coexist at fine spatial scales. In the hot springs of Lemonade Creek in Yellowstone National Park (USA), *C. merolae* (10D‐like) and *Galdieria yellowstonensis* (previously *G. sulphuraria*, resembling the strain 5587.1) co‐exist in relatively high abundance across creek biofilm, neighboring acidic soil, and endolithic microhabitats (Skorupa *et al*., 2013). Yet these species have drastically different genome organization, GC content, and trophic preferences. In addition, they are exposed to pathogens as environmental sample sequencing revealed sequences from (giant) viruses (Felipe Benites *et al*., 2024). While bacterial pathogens infecting the algae have not been identified yet, several heterotrophic unicellular eukaryotes feeding on Cyanidiophyceae have been uncovered (Reeder *et al*., 2015; Sunada *et al*., 2025). This algal assemblage underpins an ecosystem in which organisms from all domains of life cooperate at the community level, driven by photosynthetic output primarily from *C. merolae*, gene sharing via HGT, and other adaptive mechanisms that lead to complex biotic interactions (Van Etten *et al*., 2023; Stephens *et al*., 2024) and enable life in these hostile environments.

**Fig. 3:**
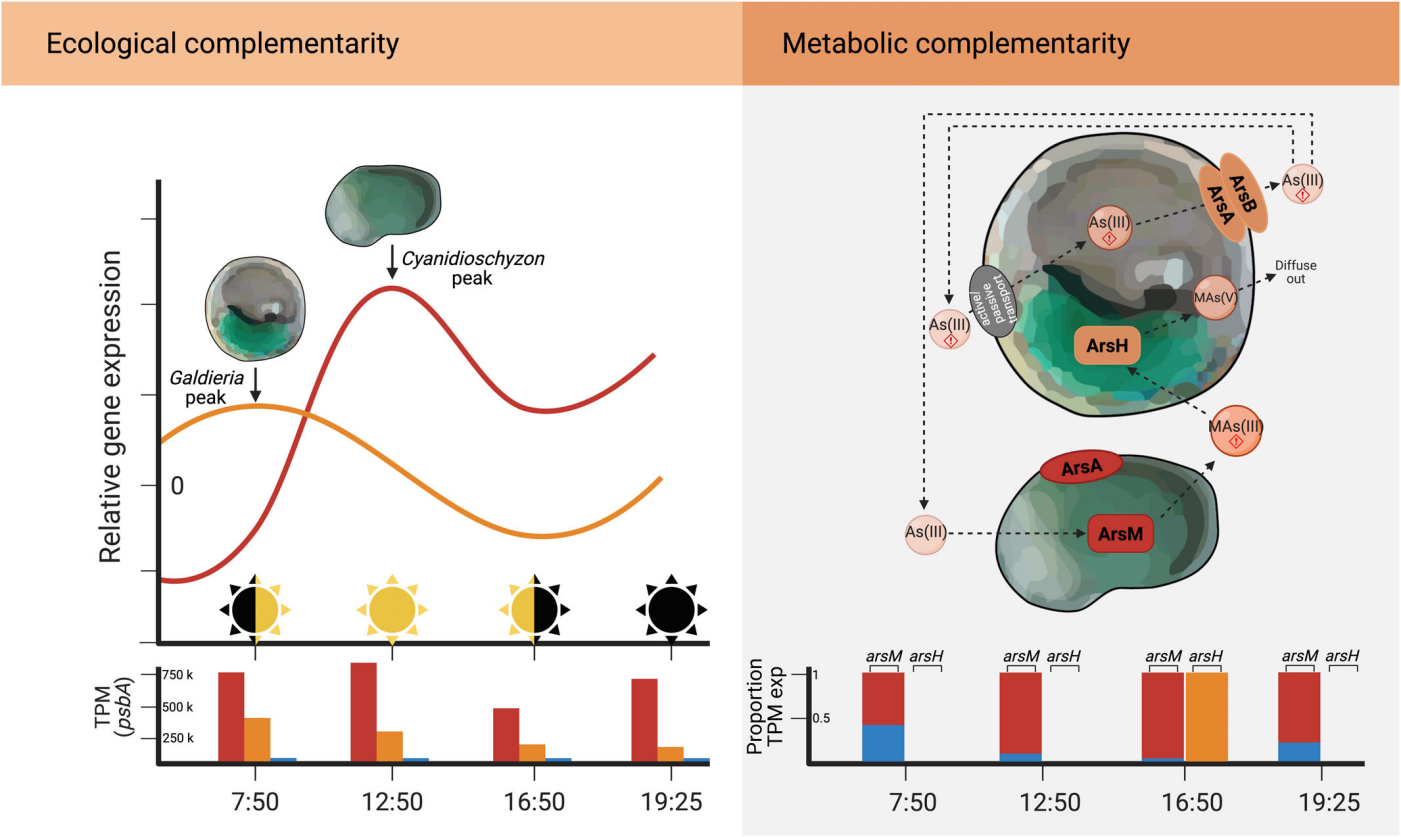
Ecological and metabolic interrelationships in the creek biofilm habitat at Lemonade Creek, Yellowstone National Park (USA). (a) Ecological complementarity: schematic representation of relative expression values of plastid genes over the diurnal cycle for *Galdieria* and *Cyanidioschyzon*. The bar graphs below show the transcripts per million (TPM) values for plastid‐encoded *psbA* for these algae, which is a marker for photosynthetic activity. *Cyanidioschyzon* is the dominant primary producer at Lemonade Creek (Stephens *et al*., 2024). (b) Metabolic complementarity: a schematic *Galdieria* cell above a *Cyanidioschyzon* cell, showing the major genes each encode for arsenic detoxification. The two possible detoxification pathways for *Galdieria* (extrusion, detoxification) are shown. The diurnal expression patterns in TPM of *arsM* and *arsH* genes at Lemonade Creek are summarized below, with *Galdieria* in orange, *Cyanidioschyzon* in red, and bacteria in blue. In this pathway, *arsM* and *arsH* theoretically work in tandem to convert arsenite to the more toxic methylarsenite and then to the less toxic methylarsenate. Although *Galdieria* can survive in the presence of arsenic in culture (Van Etten *et al*., 2024), it only expresses the *arsH* gene to a detectable level at day's end in nature (Stephens *et al*., 2024). *Cyanidioschyzon* dominates *arsM* expression throughout the day. Images were made in Biorender (https://BioRender.com/l5zy4nt).

Patterns in metagenome, metatranscriptome, and metabolome data over the diurnal cycle in Lemonade Creek show that *C. merolae* is numerically abundant and most transcriptionally active during the day (Fig. 3, Ecological complementarity) generating photosynthates that are consumed by the rest of the microbial community (Stephens *et al*., 2024). *Galdieria yellowstonensis* is most transcriptionally active at night, likely employing its ability to live as a facultative heterotroph, allowing it to take on a scavenging role. This metabolic complementarity explains the coexistence of both algae in this environment and likely explains similar patterns in other geothermal environments like Yangmingshan National Park in Taiwan (Hsieh *et al*., 2015; Liu *et al*., 2020) and Sai‐no‐Kawara Park in Kusatsu hot spring, Japan; the latter is dominated by *Cyanidiococcus* species (sister to *C. merolae*) with some *G. partita* also present.

The Cyanidiophyceae are well known for harboring a diverse assortment of adaptive HGTs of prokaryotic origin in their genomes, making up *c*. 1% of their gene inventory (Qiu *et al*., 2013; Schönknecht *et al*., 2013; Rossoni *et al*., 2019a; Cho *et al*., 2023). These HGTs originated in extremophilic prokaryotes and appear to be ancient acquisitions in the common ancestors of these algal lineages, as demonstrated by their distribution patterns among extant taxa (Rossoni *et al*., 2019a; Cho *et al*., 2023). These foreign genes confer salt and osmotic tolerance, carbon and amino acid metabolism, cellular oxidant reduction, and heavy metal resistance and detoxification. By distributing these adaptive functions across domains of life within a single extreme environment, functional redundancies and dependencies are created within populations and communities that provide potential for the evolution of new functions despite selection favoring genome streamlining (Morris *et al*., 2012; Takeuchi *et al*., 2024; Van Etten *et al*., 2024). Using arsenic detoxification as a model for the fates of HGTs, studies have shown how sharing of gene functions by members of the microbial assemblage (Fig. 3, metabolic complementarity) weakened selection on homologs in Cyanidiophyceae, allowing their long‐term persistence via the putative gain of novel functions (Cho *et al*., 2023; Van Etten *et al*., 2024). This hypothesis, called the integrated horizontal gene transfer (IHGT) model, can be used to explain broader phenomena and how extremophilic eukaryotes transitioned from their ‘hot start’ milieu by functional innovations driven by the duplication and divergence of genes that originated via IHGT (Van Etten *et al*., 2024). Here, we see differences in the retention and transfer patterns of heavy metal‐related HGTs in *C. merolae* and *G. yellowstonensis* that reflect the gain of new functions, interesting gene retention patterns, and connectivity to diverse (nondetoxification) metabolic pathways. Furthermore, recent work has shown connections between co‐occurring microbes across all domains (and viruses) based on shared DNA that may reflect past ecological connections or lay the foundation for new organismal associations (Van Etten *et al*., 2025). These results underline the need for *in situ* studies to gain meaningful evolutionary and ecological insights into ecosystem maintenance (Box 1).

## V. Importance of Cyanidiophyceae in biotechnology

**Fig. 4:**
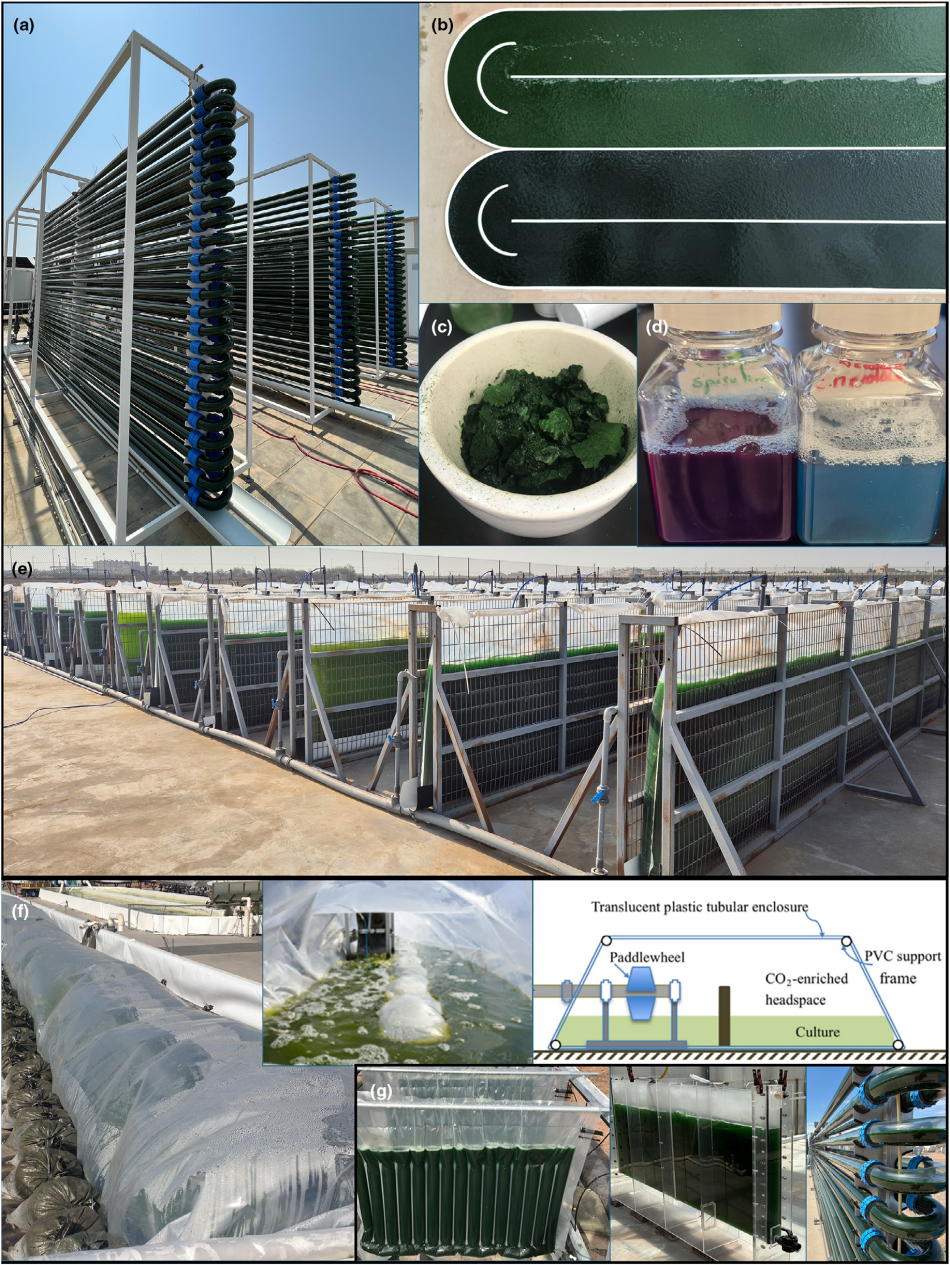
The Cyanidiophyceae can be cultivated in extreme environments for its protein‐rich biomass, C‐phycocyanin (C‐PC), and wastewater treatment. (a–e) Various photographs from the Development of Algae Biotechnology in the Kingdom of Saudi Arabia (DABKSA) project funded by the Ministry of Environment Water and Agriculture (MEWA) at King Abdullah University of Science and Technology (KAUST) from 2022 to 2025. Photos graciously provided by Dr Claudio Grünewald. (a) 3× 1000 l Varicon Aqua tubular photobioreactors growing *Cyanidioschyzon merolae* in August 2022 as previously published (Villegas‐Valencia *et al*., 2023, used under the terms of CC‐BY 4.0). (b) 2× 80 000 l raceway ponds containing *C. merolae*; upper, recently inoculated; lower, mature culture. (c) Freeze‐dried flakes *C. merolae* biomass before milling. (d) Comparison of the colour of water‐soluble C‐PC extracts from Spirulina (*Limnospira*) (left) and *C. merolae* (right) which appears lighter blue and with less other chromophores. (e) Cultivation of 125 000 l of *C. merolae* in flat panel reactors using sparged industrial green‐CO_2_ in air mixtures. Cultivations in (b) and (e) were performed by Diedrich Vahrenkamp. (f) Plastic bag enclosed raceway system for cultivation of *Galdieria* in primary settled wastewaters with diagram from (Henkanatte‐Gedera *et al*., 2015, 2017, used with permission). (g) Various styles of *Galdieria* cultivation, hanging bags, acrylic panel, and LGEM tubular photobioreactor system. (f, g) Photos provided by Prof. Peter Lammers from Arizona State University (ASU) Arizona Center for Algae Technology and Innovation (AzCATI).

The application of algae in biotechnology, in principle, uses the microbes' inherent abilities to convert inorganic chemicals (ammonia, phosphate, and carbon dioxide) into organic chemical value (protein, carbohydrates, lipids, and pigments) in their biomass, generally driven through photosynthesis as an energy source (Zhang *et al*., 2022). Their cultivation can also be used to remediate wastewaters as the primary goal, with biomasses being a valuable process output (Chen *et al*., 2018; Nagarajan *et al*., 2020). Traditional algal biotechnology applies microbial cultivation concepts to unicellular algae by enabling photosynthesis‐driven cultivation in photobioreactors (Fig. 4a,b). Some algae can be cultivated in fermentation infrastructure using organic carbon as a feedstock (Lu *et al*., 2021; Bürck *et al*., 2024). After sufficient growth, the microbial broth is harvested and dewatered, resulting in a powdered biomass product that can be used for various applications (Fig. 4c) (Fabris *et al*., 2020; Torres‐Tiji & Posewitz, 2025). Algal biomass can be a feed additive for animals, a source of specific macromolecules, or specialty chemicals. Each algal species generates a biochemically unique biomass, containing a variable content of macromolecules.

The ability of Cyanidiophyceae to thrive in high‐temperatures and low‐pH environments presents many opportunities for the biotechnologist to use these organisms in conversion of low‐value wastes into higher‐order biochemical outputs (Gross, 2000; Toplin *et al*., 2008). Their high temperature tolerance opens cultivation potentials to situations that are unsuited for other algae, like geographies with naturally high heat, or industrial facilities. Reducing the need for cooling in large‐scale microbial cultivation can also greatly reduce process costs (Novoveská *et al*., 2023). The preference of Cyanidiophyceae for low pH opens possibilities of using unconventional acidic waste (water) streams as culture media, permits direct gassing with pure CO_2_ for carbon delivery, and use of high ammonium concentrations (Hirooka *et al*., 2022; Villegas‐Valencia *et al*., 2023; Masson *et al*., 2025), all of which would cause classical algal cultures to acidify and crash. The highest growth rates are observed with high nitrogen‐to‐carbon ratios (Masson *et al*., 2025) which align well with the composition of certain industrial effluents that could be used as media (Curien *et al*., 2021) enabling waste valorization (Russo *et al*., 2021). The natural biomass compositions of the Cyanidiophyceae have value as both sources of protein and specialty chemicals (Villegas‐Valencia *et al*., 2023). These algae retain the cyanobacterial water‐soluble, blue‐colored protein complex C‐phycocyanin (C‐PC) phycobiliproteins as part of their photosystems (Yoshida *et al*., 2021). C‐PC is used as a natural food colorant and normally sourced from the alkaliphile *Limnospira* sp. (formerly *Arthrospira* or colloquially ‘Spirulina’). However, Cyanidiophyceae C‐PC is thermo‐ and acid‐tolerant, enabling pasteurization and increasing its applicability in beverages and food industries (Rahman *et al*., 2017; Masson *et al*., 2025). The US FDA recently approved C‐PC from *Galdieria* sp. as an ingredient. In organisms like *Galdieria* spp. and *C. merolae*, C‐PC accumulates to *c*. 3–5% of the cell mass and is part of the water‐soluble protein fraction, facilitating use of the blue color with minimal purification (Yoshida *et al*., 2021). Many different strains of *Galdieria* exist, with some exhibiting variability in pigment responses when organic carbon is present in the medium (Masson *et al*., 2025). The biomass of *Galdieria* and *Cyanidioschyzon* are proteinaceous (> 60%), having low triacylglycerol accumulation (<30%) (Muppaneni *et al*., 2017). Cyanidiophyceae lack alpha‐carotenoids and xanthophyll pigments, exhibiting a minimal carotenoid profile starting from beta‐carotene, with zeaxanthin as the terminal carotenoid. Both these carotenoids have value as provitamins that support macular health (Cunningham *et al*., 2007).

Recent efforts have demonstrated nuclear genome genetic engineering for carotenoid pathway modification, isoprene biosynthesis, and fatty acid tailoring (Sumiya *et al*., 2015; Seger *et al*., 2023; Villegas‐Valencia *et al*., 2025). Given its high protein content biomass composition, *C. merolae* could be a promising candidate for conversion of cellular amino acids into tailored chemicals, such as diamines or tyrosine‐derivatives like coumaric acid, melanin, or even vanillin. The feasibility of engineering recombinant protein accumulation or secretion remains open questions in this host (Box 1). Currently, no applications exist for certifying its biomass as a safe food ingredient; however, it could be as valuable as *G. sulphuraria* in both C‐PC and protein‐rich biomass production.

Together, these genera exemplify the potential of Cyanidiophyceae as robust platforms for new and expanding industrial biotechnology applications. Their unique physiological traits and genetic tractability position them as valuable tools for conducting sustainable resource recovery in existing waste streams. Their native environments are on the extreme edges of eukaryotic life, and they exhibit high tolerance to agents like dissolved metals and strong acidic conditions (Kharel *et al*., 2023). Industrial sites that already have effluents or that process residuals with these properties could be new avenues to embark on algal‐biotechnology applications using Cyanidiophyceae. For example, acidic mine process wastewater, high‐ammonia content municipal wastewater concentrates, or brewery process wastes are all examples where a Cyanidiophyceae bioprocess could be applied. However, it has been observed in many groups (not shown) that fungal contaminations are common if cultures are lower than 42°C, presenting also the challenge that processes must be built to accommodate thermal regulation. *Galdieria* spp. present the possibility of converting waste organic carbon products into proteinaceous biomass efficiently, whereas CO_2_ would be the preferred carbon source for *C. merolae*, which could be used to valorize industrial process gas emissions. An example of combining extreme Cyanidiophyceae biotechnology to new applications is the growth of *G. sulphuraria* biomass from primary municipal effluent, and the mixture of its biomass with animal manure to then conduct hydrothermal liquefaction, and application of the resulting hydrocarbon fraction as a binder in road asphalt (Pahlavan *et al*., 2020).

## VI. Molecular tools

Haploid *G. partita* does not possess a cell wall, enabling DNA delivery and genetic transformation (Hirooka *et al*., 2022), which will enable further studies of this unique order of Cyanidiophyceae; but most molecular tools have been developed for the unicellular, acidothermophilic red alga *C. merolae* (Villegas‐Valencia *et al*., 2025). The development of molecular tools was facilitated by ease of culturing (Suzuki *et al*., 1994) and transformation (Ohnuma *et al*., 2008; Villegas‐Valencia *et al*., 2025). Cell division can be synchronized by alternating light and dark cycles (Suzuki *et al*., 1994). Under optimal conditions (42°C, pH 1–3, 2% CO_2_, ≥ 90 μmol light), the doubling time is *c*. 9 h, and transformants can be isolated in < 2 wk (Villegas‐Valencia *et al*., 2025). Being haploid and having no cell wall, *C. merolae* strain 10D allows facile gene modification, deletion, and replacement using selectable markers (Fujiwara *et al*., 2013a, 2017, 2021; Taki *et al*., 2015; Zienkiewicz *et al*., 2017). Episomal DNA (plasmids) is reported to be stably maintained under selection (Ohnuma *et al*., 2008, 2009). For integration of transgenes, the endogenous homologous recombination machinery (Fujiwara *et al*., 2017) shows efficiencies of 50–100% depending on the marker and target locus (Villegas‐Valencia *et al*., 2025). Counterselectable markers such as URA5.3 can be removed if flanked by tandem repeats, allowing marker recycling (Takemura *et al*., 2018). Most transgenic insertions to date have been targeted to the CMD184/185 intergenic locus that minimally disrupts transcription and has no discernible effect on growth (Fujiwara *et al*., 2013a).

The ability to modify the genome and introduce novel sequences into cells has accelerated the development of a variety of analytical and cell biological techniques. The most straightforward modification is gene deletion, reported most notably in the case of the URA5.3 gene, which resulted in the useful URA auxotrophic strain T1 (Taki *et al*., 2015). Although *C. merolae* promoters have not yet been fully defined, transgene expression can be driven by strong endogenous promoter regions (usually 500 bp upstream of a gene) such as APCCp and CPCCp (Sumiya *et al*., 2014; Fujiwara *et al*., 2021), inducible endogenous promoters from the heat shock response pathway (Sumiya *et al*., 2014), and repressible promoters from the nitrate‐response pathway. It is thought to be important to use different promoters for each transgene to avoid recombination between multiple copies of the same promoter, given the high rates of recombination in *C. merolae*. Transcription termination is generally achieved using the bacterial NOS terminator (Sumiya *et al*., 2014). A recent study isolated plastids from *C. merolae*, owing to its easy lysis and demonstrated these could, for some time, remain viable within intracellular vesicles in mammalian cells.

Genetic engineering in *C. merolae* has made use of protein tags and various expression reduction techniques (‘knock‐downs’) at the DNA, RNA, and protein level. Protein tags for immunoprecipitation and fluorescence microscopy that have been used in *C. merolae* include fluorescent proteins, His, protein A, and StrepII (Ohnuma *et al*., 2009; Villegas‐Valencia *et al*., 2025), leading to an abundance of cell biological investigations of organelle division (Fujiwara *et al*., 2013b; Ichinose & Iwane, 2021). 2′Omethyl antisense oligonucleotides have been used successfully to isolate RNP particles from *C. merolae* whole cell extract. Antisense knockdown of transcription has been reported (Ohnuma *et al*., 2009), as has rapamycin‐induced protein degradation (Fujiwara *et al*., 2024). *Cyanidioschyzon merolae* cells are readily fixed and preserved for fluorescence microscopy and other imaging techniques, including immuno‐electron microscopy (Nishida *et al*., 2003). Finally, there exist many publications reporting transcriptomic analysis of *C. merolae* cells under a variety of different light conditions, CO_2_ levels, nutrient depletion, temperature changes, and so on (Fujiwara *et al*., 2009; Imamura *et al*., 2015; Tardu *et al*., 2016; Rademacher *et al*., 2017). These treatments enable detailed analysis of gene regulatory pathways in this organism. Recently, transcriptomic analysis has been extended to Ribo‐seq identification of actively translating transcripts in stationary and dividing cells (Mogi *et al*., 2025). These techniques have been brought together into a method that simultaneously generates tagged genes and gene deletions (Bora & Tanaka, 2025). Having generated valuable strains with gene deletions, gene modifications, transgenes, and so on, the strains can either be preserved in tissue culture flasks at room temperature and ambient light, or cryopreserved in DMSO (Ohnuma *et al*., 2011).

## VII. Molecular responses to stress in Cyanidiophyceae

Organisms can react to stressful environments by basal tolerance mechanisms where the transcriptome and proteome are reshaped after exposure to the stress and can ‘memorize’ previous stress responses to acquire an enhanced stress tolerance for recurring stress (Hilker *et al*., 2016). The latter has not been shown yet for Cyanidiophyceae (https://refubium.fu‐berlin.de/handle/fub188/37462); however, the red seaweed *Bangia fuscopurpurea* can acquire memory of sublethal high‐temperature stress, resulting in acquired thermotolerance (Kishimoto *et al*., 2019).

*Cyanidioschyzon merolae* optimally grows at 42°C and can survive temperatures up to 60°C. Interestingly, heat shock genes are induced at an exact temperature suggesting a thermal sensing mechanism, which likely activates heat‐protecting mechanisms (Kobayashi *et al*., 2014; Sumiya *et al*., 2014). Two of the highly induced genes encode small heat shock proteins (sHSP). These two genes share a common promoter and are highly similar; however, the proteins are predicted to be nuclear and localized to chloroplasts, respectively, suggesting a protective function for the different organelles (Kobayashi *et al*., 2014). *Galdieria sulphuraria* has a similar temperature optimum as *C. merolae*, and reduction in growth temperature from 42°C to 28°C resulted in massive transcriptional changes, including an upregulation of genes involved in protein biosynthesis. While the transcription factors involved in cold responses in higher plants are not conserved in Cyanidiophyceae, *G. sulphuraria* shows metabolic acclimation to cold stress by synthesis of betaines, which some nonthermophilic organisms also use to acclimate to lower temperatures (Rossoni *et al*., 2019b).

The responses of Cyanidiophyceae to light conditions vary strongly as their natural habitats range from very low light intensity (e.g. in endolithic habitats) to a high light level for species growing in streams and open ponds. Under high light conditions, reactive oxygen species accumulate and need to be detoxified which is at least partially achieved by ascorbic acid and the activity of ascorbate peroxidases (Fu & Wang, 2023), revealing a conserved mechanisms between algae and higher plants (Yoshimura & Ishikawa, 2024).

Chromatin proteins protect and compact the DNA and influence the expression of the genetic information (Kouzarides, 2007; Li *et al*., 2007). Covalent modification of chromatin proteins and DNA forms an ‘epigenetic’ information that can be maintained through cell division. This epigenetic information acts as a memory and plays a role in the response to stress in eukaryotes (Ma *et al*., 2025). The basic unit of eukaryotic chromatin is the nucleosome, comprising two copies of each core histone: two heterodimers of H2A and H2B and one tetramer of H3 and H4. Each of the core histones H2A, H2B, and H3 is strongly diversified in land plants and mammals (Loppin & Berger, 2020; Martire & Banaszynski, 2020). Although H3 and H4 are > 95% conserved among red algae and green plants, H2A showed marked differences. Remarkably, H2A.X is absent in *C. merolae*, whereas H2A.Z is absent in several isolates of *Galdieria*, which is truly exceptional, since the complete loss of H2A.Z is nonviable in most eukaryotes. Interestingly, the H2A sequences in *C. merolae* and *Galdieria* are very distinct not only from each other but also from other nonthermoacidophilic species. Through their different combinations, histone variants provide a code across the genome that helps to regulate gene expression (Bönisch & Hake, 2012; Loppin & Berger, 2020). For example, in the flowering plant *Arabidopsis thaliana*, H2A.Z associates with H3K27me3 to repress protein‐coding genes, whereas the histone variant H2A.W associates with H3K9me2 and DNA methylation to repress transposons (Jamge *et al*., 2023). Transposons in turn have the capacity to carry the epigenetic mark and confer a stress‐sensitive regulation to genes in proximity of their insertion (Thieme *et al*., 2022). Although H2A.W and DNA methylation are absent in *C. merolae*, H3K27me3 covers transposons and causes their silencing (Mikulski *et al*., 2017; Hisanaga *et al*., 2023). This highlights an ancestral role of this epigenetic mark also found in several other protists (Hisanaga *et al*., 2023). Although much work remains to be done on the role of chromatin in Cyanidiophyceae, the first studies suggest that together with works in a broader range of eukaryotes (Grau‐Bové *et al*., 2022; Romero Charria *et al*., 2025), red algae will provide a glimpse into the ancestral pattern of chromatin regulation in eukaryotes and their roles in adaptation.

## VIII. Conclusions

Cyanidiophyceae possess sophisticated stress response systems that enable survival in environments that are lethal to most eukaryotes. These species provide insights into both highly conserved stress resistance mechanisms and the importance of HGT in acquiring novel stress response traits. Whereas extensive genomic resources are now available, comprehensive analyses of transcriptomic, proteomic, and metabolomic responses to diverse stressors are still lacking. In particular, the genetic identification of master regulators governing stress resilience remains a key research gap. *Cyanidioschyzon merolae*, with its highly reduced genome and available genetic tools, is well suited for such investigations, whereas *Galdieria* species offer insights into metabolic flexibility and the acquisition of new traits via HGT. The significance of mechanistic studies will be illuminated by a deeper understanding of the ecology of Cyanidiophyceae. To differentiate the particular from general features it will be crucial to place studies in these fascinating organisms in the broader context of the biology and evolution of algae (Goldbecker & de Vries, 2025; Kunz *et al*., 2025) and more specifically of red algae. This will now be possible with advances in genomics and establishment of the first model multicellular alga (Petroll *et al*., 2025).

## Competing interests

None declared.

## Author contributions

Each author contributed to a section of the text. FB coordinated the project and edited the manuscript.

## Disclaimer

The New Phytologist Foundation remains neutral with regard to jurisdictional claims in maps and in any institutional affiliations.

## Supporting information


**Fig. S1** Diversity of Cyanidiophyceae microhabitats and allochthonous carbon sources at GenZiPeng geothermal area, Taiwan.
**Fig. S2** Comparative physiological responses of *G. partita* and *C. yangmingshanensis*.
Please note: Wiley is not responsible for the content or functionality of any Supporting Information supplied by the authors. Any queries (other than missing material) should be directed to the *New Phytologist* Central Office.
