# Supplementary material for: Fascinating single‐cell red algae: models for evolution and adaptation
Source: New Phytol. 2026 Feb 18;250(3):1424–37. doi: 10.1111/nph.71024 (PMC13062707; doi:10.1111/nph.71024)

New
Phytologist

New Phytologist Supporting Information

Article title: **Fascinating single cell red algae: models for evolution and adaptation**

Authors: Frédéric Berger, Debashish Bhattacharya, Chung Hyun Cho, Seok-Wan Choi, Julia Van Etten, Shunsuke Hirooka, Tzu-Yen Huang, Kyle J. Lauersen, Yongsung Lee, Shao-Lun Liu, Shin-ya Miyagishima, Stephen D. Rader, Daniel Schubert, Hwan Su Yoon

Article acceptance date: 20 January 2026

Fig. S1 Diversity of Cyanidiophyceae microhabitats and allochthonous carbon sources at GenZiPeng geothermal area, Taiwan. (a) Landscape showing aquatic (black arrow) and non-aquatic (white arrow) sites. (b) Sunlit geothermal stream with submerged algal mats typical of aquatic habitats. (c) Cyanidiophyceae growing on acidic soil in a shaded, non-aquatic site. (d) Sulfur vent in a non-aquatic zone with visible algae and sulfur deposits. (e-f) Endolithic Cyanidiophyceae biofilms in less weathered rock with shallow biofilm (e) and more weathered rock with deeper biofilm (f), showing the avoidance of strong light. (g) Epilithic Cyanidiophyceae biofilm on a shaded rock surface. (h) Shaded non-aquatic site with decaying plants and adjacent Cyanidiophyceae growth (inset). (i) Moss-covered bank near a geothermal stream with a resting spider (inset). (j) Carcass of dead black kite in a non-aquatic site with decomposing tissue as a possible organic matter source (inset).

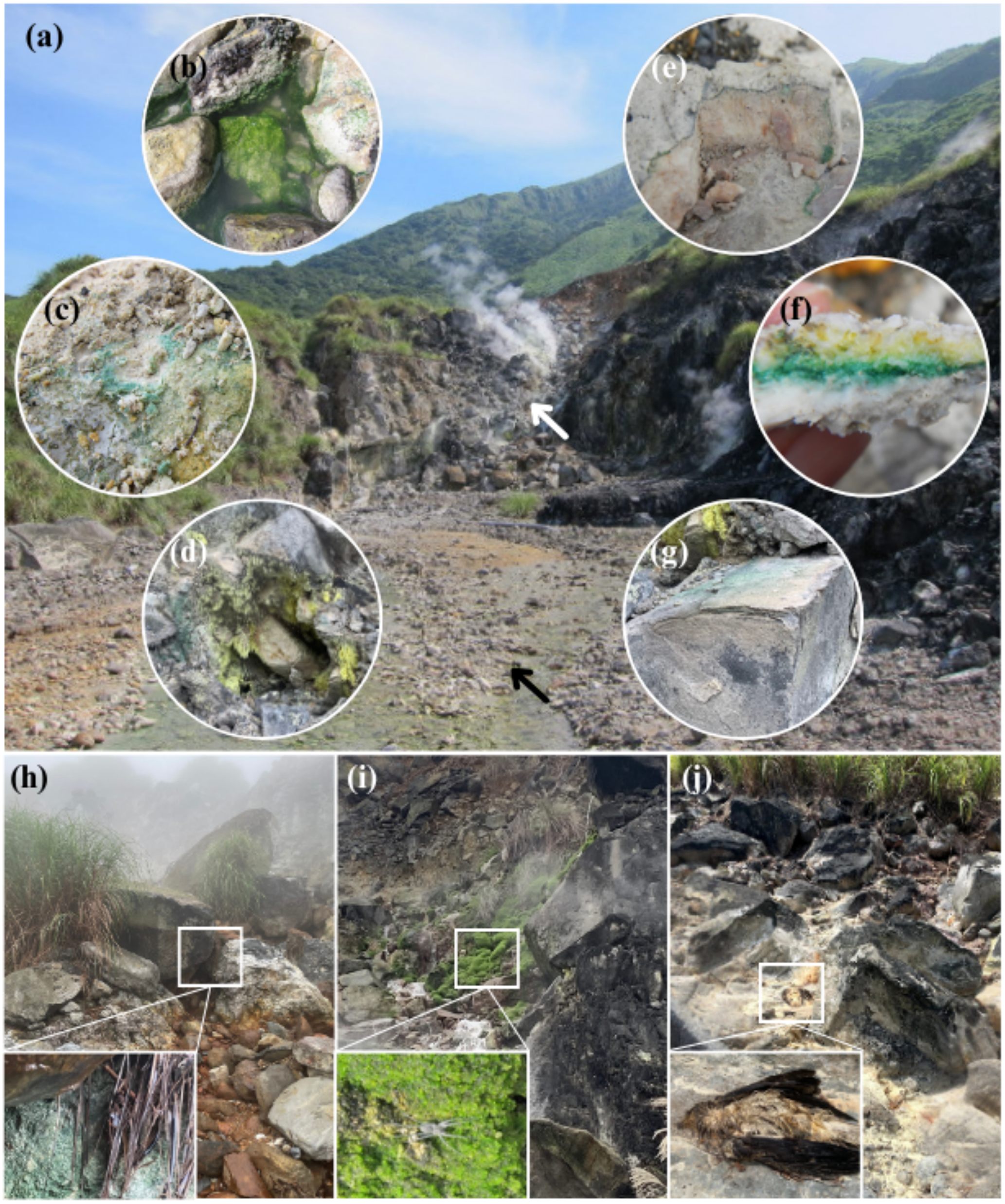

Fig. S2 Comparative physiological responses of *G. partita* and *C. yangmingshanensis* (see the experimental details in the Materials and Methods. Growth under increasing PEG 6000 concentrations simulating low water potential (i.e., drier conditions). Different letters denote significant differences ($p < 0.05$).

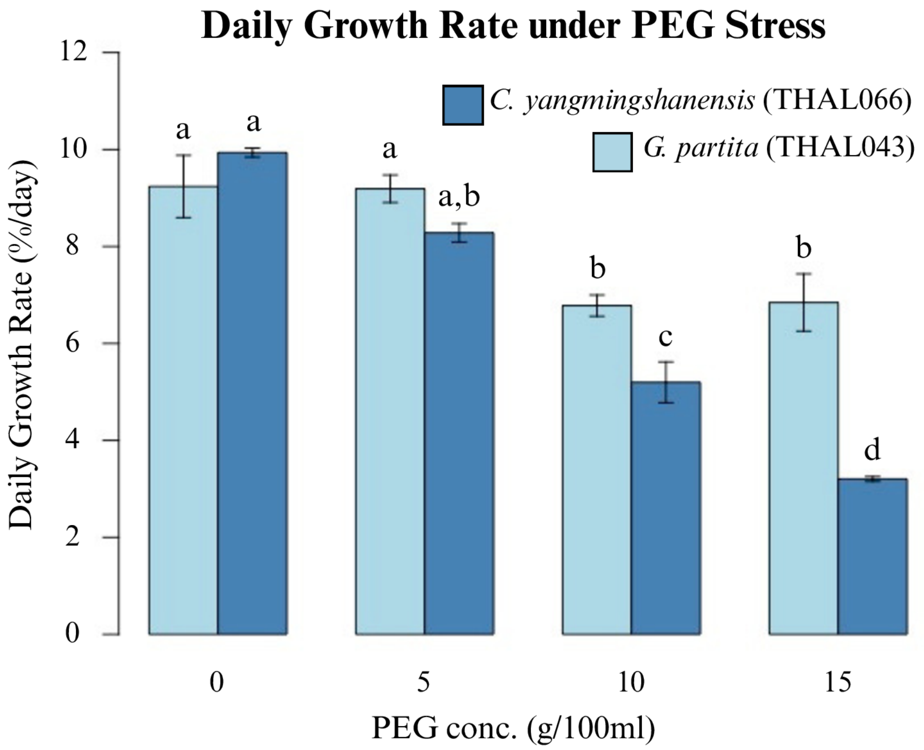

Supplement: Supplementary file 1 — Fig. S1 Diversity of Cyanidiophyceae microhabitats and allochthonous carbon sources at GenZiPeng geothermal area, Taiwan. Fig. S2 Comparative physiological responses of G. partita and C. yangmingshanensis. Please note: Wiley is not responsible for the content or functionality of any Supporting Information supplied by the authors. Any queries (other than missing material) should be directed to the New Phytologist Central Office. [file NPH-250-1424-s001.pdf]
